# Exploring the Potential of Exogenous Dietary Lysozyme as a Bioactive Additive in Aquaculture: Lessons from Monogastric Livestock Nutrition

**DOI:** 10.1007/s12602-026-10933-y

**Published:** 2026-02-21

**Authors:** Christina Zantioti, Emmanouil E. Malandrakis

**Affiliations:** Laboratory of Applied Hydrobiology, Faculty of Animal Science, Agricultural University of Athens, Iera Odos 75, Athens, 11855 Greece

**Keywords:** Aquaculture, Exogenous enzymes, Dietary enzymes, Lysozyme, Dietary lysozyme

## Abstract

The growing demand for sustainable aquaculture practices underscores the need for innovative solutions that not only improve health and performance but also enhance disease resistance. Lysozyme is an enzyme with antimicrobial activity that can be found in various tissues. Its immunomodulatory properties have been shown to have promising results in terrestrial animals, particularly enhancing growth performance, gut health and immune response. Nevertheless, its application in aquaculture remains underexplored. This review compiles current existing evidence on dietary supplementation of exogenous lysozyme in aquatic animals, drawing insights from studies in monogastric livestock. Additionally, it collects the limited available evidence regarding the use of exogenous lysozyme in fish and shrimp. Finally, this review highlights the potential limitations related to the use of lysozyme as a functional additive in aquafeeds.

## Introduction

 Aquaculture offers a sustainable source of healthy, nutritious food, which is urgently needed for the transformation of global food systems [1]. According to FAO, with a growth of 3.5% in the 2020 s, aquaculture is predicted to provide sufficient food, nutrition and livelihoods for the world population that is projected to reach 8.5 billion in 2030 [2]. Nevertheless, despite its potential, intensive aquaculture still faces challenges such as poor growth performance and increased susceptibility to infectious diseases [3]. These challenges are reflected in recent FAO recommendations calling for strengthened diagnostic capacity, improved disease prevention, enhanced biosecurity practices, and greater cooperation among states to manage transboundary aquatic animal diseases. A lack of consistent implementation of such measures continues to expose aquaculture systems to recurrent health risks [4] Specifically, injudicious use and/or misuse of antimicrobial agents to combat diseases of reared species poses a significant threat to the development and the sustainability of the aquaculture sector [5]. In line with these concerns, suggestions have been made to reduce antimicrobial use in aquaculture, develop national antimicrobial resistance action plans, and prioritize alternatives such as vaccines and immunostimulants. These recommendations highlight a global shift toward more sustainable health management strategies and the urgent need for effective non-antibiotic interventions [4]. Thus, it becomes evident that there is an urgent need to address such challenges with the adoption of innovative approaches. One potential solution is the use of novel additives which can not only enhance the performance of aquatic animals but also act as immunostimulants boosting their defense mechanisms and improving resistance to diseases [6].

Functional additives are dietary ingredients that are incorporated in feed formulations, not only to meet basic nutritional requirements but also to enhance growth, health and overall production efficiency [7]. Lysozyme is a natural peptide with antibacterial and immunomodulatory properties [3]. It exhibits strong antibacterial activity through its ability to hydrolyze the β−1,4 glycosidic bonds in bacterial cell walls. Because of this mode of action, it has been widely applied in the food and pharmaceutical industries as a natural antimicrobial agent [8]. Recently, its antimicrobial and immunomodulatory properties have also attracted interest in the feed industry as a potential functional additive. While there are studies available showing the growth-enhancing and immune-enhancing potential of lysozyme, the majority of this research focuses on terrestrial animals.

In monogastric livestock like pigs and poultry, lysozyme supplementation has been shown to improve growth, gut health, and immune responses, providing valuable insights into its potential mechanisms of action. To our knowledge, few studies have specifically investigated its role in aquaculture. While acknowledging that fish differ from monogastrics in several physiological aspects, comparative findings from terrestrial animals still offer a useful biological framework for exploring lysozyme’s relevance in aquaculture. Therefore, this review aims to examine the role of exogenous dietary lysozyme in aquatic animals by compiling the current knowledge in aquaculture, while also highlighting relevant insights drawn from nutritional studies on monogastric livestock. Through this approach, we aim to provide conceptual, translational insights and practical relevance of lysozyme as a potential feed additive for aquaculture.

## Bioactive Additives in Aquaculture

According to the European Food Safety Authority (EFSA), zootechnical additives are additives used to favorably affect the performance of animals in good health or the environment [9]. In aquaculture, bioactive additives are used to support animals’ health by contributing through various mechanisms, including antimicrobial, antioxidant, immunostimulant, growth-enhancing, and anti-inflammatory effects [10]. Bioactive compounds can be proteins, enzymes, polysaccharides, lipids, and secondary metabolites, deriving from sustainable sources such as algae, plants or microbial biomass that are added into aquaculture feeds and/or water systems. These compounds with their direct and indirect modes of action can enhance growth performance, immunity and stress resilience in aquatic species, while also reducing environmental burdens [6, 11].

Bioactive additives offer promising new perspectives for improving animal health and performance. However, as they are complex compounds, their use requires further research to fully understand their mechanisms of action and their effects on animal physiology. For instance, understanding the intestinal microbiota, the interactions between microorganisms themselves or between them and the organs and tissues of the host can help assess the capabilities of such natural compounds [12]. Such knowledge is essential not only for optimizing their inclusion in animal diets but also for developing more targeted, sustainable, and health-oriented feeding strategies in animal production [13].

## Enzymes in Aquaculture

Nutritional interventions with exogenous enzymes have become an effective way to improve the performance of aquaculture species. Usually, enzymatic supplementation is used to improve feed utilisation and nutrient digestibility, enhance growth performance, optimise whole-body composition, and promote immune function and resistance to pathogens. Additionally, enzymes are used to modulate the gut microbiota of fish. Exogenous enzymes include lipases, chitinolytic enzymes or carbohydrate-digesting enzymes like α-amylase [14]. Inclusion of protease has been shown to improve protein digestibility in Gibel carp (*Carassius auratus gibelio*), Caspian salmon (*Salmo trutta caspius*) and tilapia species [15]. Amylase is also known to enhance metabolic activity and regulate blood glucose levels. Studies using α-amylase in *Labeo rohita* diets have demonstrated these effects. Amylase addition in striped catfish (*Pangasianodon hypophthalmus*) feeds exhibited immune activity, improving red and white blood cells count, hematocrit, and the numbers of lymphocytes, along with other haematological parameters [16]. Moreover, inclusion of microbial chitinases is considered a promising solution to improve nutrient uptake in European seabass (*Dicentrarchus labrax*) and other important marine species, particularly in the context of novel feed ingredients such as insect meals, which are rich in chitin and may pose a challenge to nutrient digestibility and in determining the appropriate inclusion rates [17]. Lastly, supplementation with commercial exogenous enzyme complexes increased the richness and abundance of the microbiota and enhanced the digestibility of diets rich in plant feedstuff for turbot juveniles (*Scophthalmus maximus*).

## Lysozyme

### Structure and Mechanism of Action

Lysozyme or muramidase or N-acetylmuramide glycanhydrolase is an enzyme of the innate or non-specific immune system, which is involved in the protection against bacteria as it has bacteriostatic and bactericidal action [18, 19]. It consists of 129 amino acids with four disulfide bridges linking specific regions within a single polypeptide chain [20]. Specifically, lysozyme hydrolyzes the β−1,4-glycosidic bond of the peptidoglycan found in the bacterial cell wall, leading to cell lysis [20, 21]. To do so, the enzyme forces the sugar in the muramic acid of the peptidoglycan layer of the cell wall and by the combined action of Glutamic acid 35 and Aspartic acid 52, the two key residues, it hydrolyses the glycosidic bond [22]. Lysozyme acts directly on Gram-positive bacteria, however, in the case of Gram-negative bacteria, access to the inner peptidoglycan layer requires the complementary action of other enzymes. These enzymes serve to facilitate the exposure of this inner peptidoglycan layer to lysozyme [19, 23].

Lysozyme was the first enzyme for which the connection between its structure and function was clearly established [20, 21]. It is a small alkaline molecule with an average molecular weight of 14.3 kDa [24]. The amino acids lysine and leucine are the N and C terminal of the enzyme, respectively, while its six helix regions and disulfide bridges are responsible for the enzyme’s high thermal stability. Lysozyme has a native structure of two domains: the alpha and the beta [20, 22]. The synthesis of lysozyme in fish takes place in the liver and various non-liver tissues [23].

There are three main categories of lysozyme: the “c”, the “g”, and the “i” form [24]. In fish, we can find the c- and g- types. Specifically, in species like the common carp (*Cyprinus carpio* L.), rainbow trout (*Oncorhynchus mykiss)* and Atlantic salmon (*Salmo salar*) the c-type can be found, while in species like the sea bass (*Dicentrarchus labrax*), the g-type can be found [25]. When it comes to the molecular evolution of the various types, it is suspected that the c- and g-type come from a common precursor molecule similar to the g-type lysozyme [26]. Nevertheless, the two types are considered to have great genetic differences. Fish c-type lysozyme consists of 4 exons and 3 introns, while the g-type consists of 5 exons and 4 introns, respectively. Also, g-type lysozymes tend to be heavier molecules [25].

Lysozyme can be found in almost all animal secretions, body fluids, and tissues. In fish, it is secreted by the leukocytes and exhibits systemic distribution, having been identified in mucus, lymphoid tissues, plasma, and kidneys [23, 27, 28]. Its activity can be used as an immune status indicator, as its increase indicates the presence of a high bacterial load [23, 27, 28]. An extensive review on the role of endogenous fish lysozyme and the factors that affect its activity has been conducted before [23]. Due to its characteristics, lysozyme has been explored as an alternative to antibiotics in several research studies, being used as an additive in animal feed [29]. The oral administration of lysozymes is a promising approach, as it may help inhibit the spread of gastrointestinal pathogens or modulate the composition of the entire gut microbiota [30]. However, to our knowledge, very few studies are available on its use in aquatic animals and to date, no review has been published that compiles and summarizes the available literature on the topic of exogenous lysozyme in aquafeeds.

### Categories of Lysozyme

As mentioned above, lysozyme is abundant in nature. However, not every source is suitable for large-scale industrial production of lysozyme. One of the more convenient sources of lysozyme is birds’ eggs, with hen eggs being the richest in concentration (2500–3500 µg/ml) and thus being the main source of biologically active preparations of lysozyme [24]. Although not as rich as hen-egg whites, milk also contains lysozyme [31].

Noted previously, based on characteristics like the structure, catalysis and immunization, lysozymes are divided into three main families: chicken-type (c-type), goose-type (g-type) and invertebrate-type (i-type) [32]. Human lysozyme (HL) is the first sequenced mammalian lysozyme that along with hen-egg white lysozyme (HEWL), has been widely used over the past 30 years in research. HL possess a slight advantage over HEWL as it is less likely to trigger an immune response, but as its sourcing is limited its use is restricted for industrial use [33]. For that reason, genetic engineering techniques are usually employed to produce recombinant human lysozyme (rhLZ) in various host organisms such as plants, bacteria and yeast [24, 33].

Yeast, fungi, bacteria, and phages have also been used as sources to produce lysozyme. *Pichia pastoris*, a methylotrophic yeast, is considered an effective system for the expression of heterologous proteins, and it has been used to produce lysozyme [31, 34]. *Bacillus licheniformis* from soil, *Escherichia coli* and the fungus *Chalaropsis sp.* have also been used to produce microbial lysozyme [31, 35]. A bacteriophage lysozyme has also been identified for its potential application to prevent and treat bacterial infections [31, 36].

## Paradigms from Non-Ruminant Livestock

In this review, we will focus exclusively on monogastric species, namely poultry and swine. A brief consideration of other species, including rabbits and quails, will also follow.

### Poultry

Several research studies are available on the use of microbial lysozyme in poultry production (Table 1). Lysozyme has demonstrated a favourable safety profile in poultry and has been shown to reduce the occurrence of sand-shelled eggs, particularly at dietary inclusion levels between 200 and 300 mg/kg [37]. Lysozyme supplementation to broiler chickens improved the birds’ growth performance and enhanced intestinal health through modulation of gut microbiota. The expression of IFN-γ, IL-10, and IL-18 genes in ileal samples was also enhanced when birds were fed exogenous lysozyme, with the most noticeable results observed in broiler chickens receiving 90 mg lysozyme supplementation per kg of basal diet [38]. Administration of aqueous microbial lysozyme through drinking water improved both immune and anti-inflammatory responses in birds [39]. After a 28-day feeding trial, chickens that were fed diets supplemented with 40 mg/kg lysozyme exhibited improved growth performance and a better intestinal barrier function. Lysozyme also exhibited immunomodulating activity and directly inhibited the spreading of *Clostridium perfringens* in the small intestine [40]. Moreover, at the end of a 42-day trial, supplementation with exogenous hen-egg white lysozyme (HEWL) influenced the expression of metabolism-related genes. Specifically, genes related to carbohydrate metabolic processes and carbon metabolism were significantly enriched in the cecal microbiota of chickens, indicating the role of lysozyme in potentially enhancing the capability of the cecal microbiota to use carbon sources in the digesta [41]. Addition of lysozyme mixed with EDTA altered broilers’ intestinal microbiota at 100 mg/kg inclusion [42]. Mortality due to necrotic enteritis (NE) was also reduced when broilers where supplemented lysozyme while the improved intestinal integrity of the birds [43]. Lastly, 40 mg/kg diet of lysozyme inclusion influenced the morphology of the small intestine [44].

**Table 1:** Summary of available results of dietary lysozyme applications in poultry. (*): dose expressed in mL/L water and mL/bird spray doses represent volume-based administration and are not directly comparable to mg/kg feed inclusion

Type of lysozyme	Inclusion Levels (mg/kg)	Trial Duration (days)	Main Effects	Reference
Commercial lysozyme	0, 70, 90, 120 mg/kg	35 days	Improved growth, enhanced intestinal health, positive modulation of gut microbiota, upregulated genes related to gut antioxidant status and nonspecific immunity.	[38]
Exogenous lysozyme	0, 40 mg/kg	28 days	Decreased *C. perfringens* colonization, improved intestinal health and growth	[40]
Hen-egg white lysozyme	0, 40, 100, 200 mg/kg	42 days	Enrichment of various genes, including genes involved in metabolic processes	[41]
Hen-egg white lysozyme mixed with EDTA	0, 100 mg/kg	35 days	Reduction of *E. coli* in the ileum, alterations in the intestinal microbiota	[42]
Commercial lysozyme	0,100 mg/kg	21 days	Reduced NE-associated mortality, enhanced intestinal health	[43]
Lysonir aqueous microbial lysozyme	0, 0.5 mL/L of drinking water, 6.5mL/200 bird for the coarse spray form ^*^	35 days	Improved feed efficiency, anti-inflammatory and immune response	[39]
Lysozyme (purity of 10% and an enzyme activity of 3110 U/mg)	0, 100, 200, 300, 400 mg/kg	Preliminary trial 28 days Formal trial 56 days	Reduced sand-shelled egg rate, improved intestinal morphology, immunity and digestibility	[37]
Hen-egg white lysozyme	0, 40 mg/kg	35 days	Influenced gut morphology	[44]

### Swine

Similar to poultry, there are also several studies available, involving trials in swine (Table 2). Dietary supplementation of lysozyme to weanling piglets was capable of accelerating the growth of weanling piglets, improving gut health and nonspecific immunity. Moreover, supplementation of 90 mg/kg lysozyme in diets was as effective as antibiotics in improving the growth performance of the weaning piglets [45]. Supplementation of 500 mg/kg dietary lysozyme led to a significant increase in the serum’s total protein and albumin levels [46]. A 19-day study demonstrated that 1000 mg/kg inclusion of lysozyme improved the growth performance of weaned piglets, improved their intestinal barrier function, and relieved inflammation [47]. A challenge test using *E. coli* suggested that dietary lysozyme could alleviate infection-related stress on piglets as well as reduce intestinal *E. coli* levels, white blood cell counts, and stress hormones [48]. Lysozyme inclusion on sows’ diets benefited their gut microbiota composition by reducing the richness of species like *E. coli* and *Lactobacillus amylovorus*. Moreover, inclusion of 1000 mg/kg of lysozyme upregulated microbial metabolic functions [49].

**Table 2:** Summary of available results of dietary lysozyme applications in swine

Type of lysozyme	Inclusion Levels (mg/kg)	Trial Duration (days)	Main Effects	Reference
Egg-white lysozyme	0, 30, 60, 90, 120 mg/kg	28 days	Improved growth, gut health and immunity	[45]
Lysozyme product with 10% lysozyme and 90% carrier	0, 500, 1000 mg/kg	14 days	Improved intestinal health and microbiome	[46]
Commercial lysozyme	0, 1000 mg/kg	25 days	Enhanced stress response induced by *E. coli*	[48]
Hen- egg white lysozyme	0, 500, 1000 mg/kg	21 days	Enhanced gut microbiome, serum immunity and milk composition	[49]
Commercial lysozyme	0, 1000 mg/kg	19 days	Improved growth, inflammation and gut health	[47]

### Other Species

In addition to broilers, exogenous lysozyme is also considered a highly promising dietary approach for improving growth performance and welfare of rabbits [38, 50]. Supplementation of up to 150 mg/kg of exogenous lysozyme improved performance, blood lipid profile and antioxidant status of rabbits as well as increased beneficial bacteria in the gut [50]. Dietary lysozyme also improved thyroid hormonal function, haematology and protein efficiency in male rabbits [51]. Moreover, HEWL has been shown not only to improve growth rate and feed efficiency of rabbits but also to reduce the counts of potential pathogens such as *E. coli* and *Clostridium* [52]. Quails have also been shown to benefit from dietary lysozyme supplementation. Exogenous lysozyme can boost the growth performance of quails by upregulating genes related to growth and feed intake, as well as genes related to metabolism. For the growing period, supplementation of 100 mg/kg is recommended [53], while 200 mg/kg inclusion is recommended for the laying period [53]. The addition of natural lysozyme in Japanese quails’ diet led to a modulation of superoxide dismutase (SOD), catalase (CAT), glutathione peroxidase (GPX), and interleukin-1 beta (IL-1β) gene expression levels, indicating immune and antioxidant effects [54]. Fungal lysozyme alleviated inflammation in the intestine of Dextran Sulfate Sodium-challenged mice in a dose-dependent way [55]. The in vivo anti-inflammatory effects of exogenous lysozyme were also noted in mice after lipopolysaccharide-induced systemic inflammation [56]. Table 3 summarizes the results of dietary exogenous lysozyme application on other species.

**Table 3:** Summary of available results of dietary lysozyme applications on other species. (*): doses expressed as mg/kg BW/day represent body-weight–based administration and cannot be directly converted to mg/kg feed doses

Species	Type of lysozyme	Inclusion Levels (mg/kg)	Trial Duration (days)	Main Effects	Reference
Rabbit	Commercial lysozyme	0, 50, 100, 150 mg/kg	42 days	Enhanced growth and antioxidant capacity, lowering of blood lipid profile, regulation of caecal fermentation.	[57]
	Hen egg white lysozyme	0, 50, 100, 200 mg/kg	56 days	Improved growth and feed efficiency and reduced total counts of *E.coli* and *Clostridium*	[52]
Mouse	Fungal lysozyme	0, 0.5 mg/kg	84 days	Alterations in the gut microbiome	[55]
	Hen egg white lysozyme	0, 4.5, 450, 2250 mg/kg body weight/day ^*^	7 days	Ameliorates systemic inflammation and could also have organ specific effects	[56]
Quail	Hen egg white lysozyme (HEWL) and commercial lysozyme	0, 100 mg/kg diet, 100 and 200 mg/kg	28 days	Modulation of SOD, CAT, GPX, and IL-1β gene expression	[54]

## Current Evidence in Aquaculture

When it comes to aquatic species, few studies were identified using dietary supplementation of an exogenous diet into aquaculture feeds. Table 4 summarises the current knowledge available for aquaculture-related species. Dietary supplementation of recombinant human lysozyme (rhLZ) seemed to significantly increase the number of blood cells, the total antioxidant capacity, the phagocytic activity, as well as the overall lysozyme levels of the Pacific white shrimp (*Litopenaeus vannamei*) [29]. The use of fermented microbial lysozyme also enhanced the immunity of *L. vannamei* while modulating the activity of digestive enzymes [58]. Also, inclusion 125 mg/kg of hen egg-white lysozyme (HEWL) in Pacific white shrimp diets inhibited the growth of *Vibrio* species while stimulated the expression of immune-related genes like prophenoloxidase, serine proteinase and lipopolysaccharide and β−1, 3-glucan binding protein and antioxidant related-genes such as superoxide dismutase and ferritin [59]. Moreover, it upregulated the expression of immune-related genes in the gills [29]. Inclusion of HEWL at 1000 mg/kg, significantly enhanced the SGR and the feed efficiency of *Carassius auratus gibelio* juveniles, while inclusion of 500 mg/kg improved the survival rates of the fish following a challenge test using *Aeromonas hydrophila* [60]. Similar results were also observed in trials using rainbow trout (*Oncorhynchus mykiss*). Specifically, the inclusion of HEWL, enhanced the activity of several antioxidant enzymes in the blood, while 450–600 mg/kg of lysozyme can improve growth and the non-specific immune system of the trout [61]. Furthermore, inclusion of microbial lysozyme in trout juveniles significantly improved the humoral immunity as well as the defensive mechanisms of the mucous membranes [62]. Egg white lysozyme (EWL) in inclusion levels up to 2 g/kg seemed to enhance growth performance and the immune response of Nile tilapia (*Oreochromis niloticus*). Also, up to 2000 mg/kg inclusion of HEWL increased resistance against *Aeromonas hydrophila* for up to 15 days post challenge [63]. Similarly, dietary HEWL improved the growth performance, the intestinal health and digesting enzyme activity and the resistance against *Streptococcus agalactiae* of red tilapia hybrids (*Oreochromis niloticus x O. mossambicus*) [64].

**Fig. 1:**
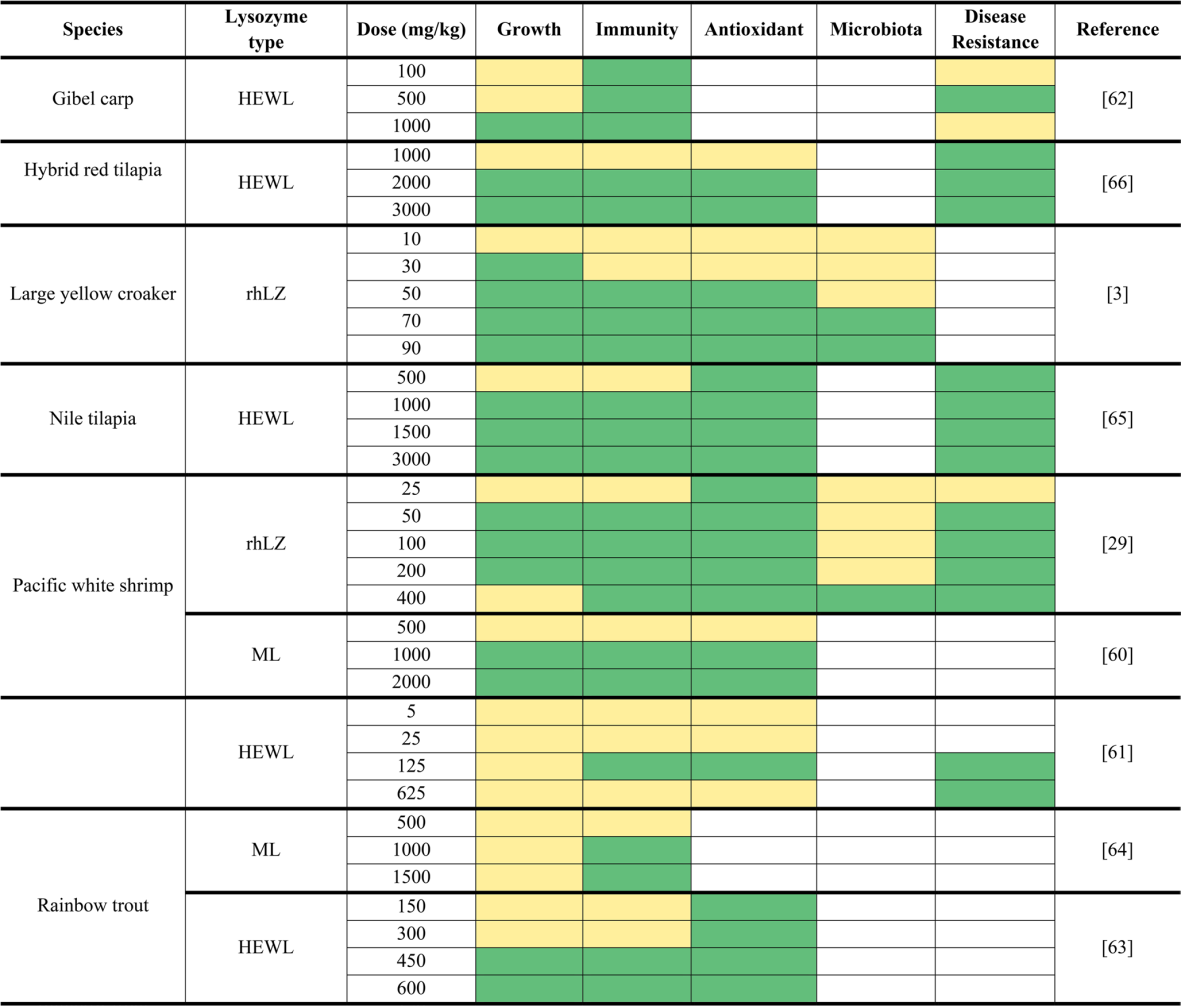
Heatmap of lysozyme effects per species, dose and type of lysozyme. Green indicates a positive effect, yellow indicates no effect, and white indicates that the effect was not studied. Lysozyme types: HEWL = hen egg-white lysozyme, ML = microbial lysozyme, rhLZ = recombinant human lysozyme

Latest trials supplementing rhLZ in large yellow croaker’s diet (*Larimichthys crocea*) suggested that the inclusion of lysozyme improved growth performance, immunity, intestinal microbiota, and muscle quality of the fish while suggesting that the optimal inclusion level is estimated to be around 67.14 mg/kg based on the quadratic regression for weight gain [3]. Figure 1, shows the dose-dependent effects of different lysozyme types on multiple aquaculture species.

**Table 4:** Summary of available results of dietary lysozyme applications in aquaculture

Species	Type of Lysozyme	Inclusion Levels (mg/kg)	Trial Duration (days)	Main Effects	Reference
Pacific white shrimp (*Litopenaeus vannamei*)	Recombinant human lysozyme	0, 25, 50, 100, 200, 400 mg/kg	56 days	Regulation of growth performance, improved digestive enzyme activity, increasing intestinal villi height and intestinal wall thickness.	[29]
Large yellow croaker (*Larimichthys crocea*)	Recombinant human lysozyme	0, 10, 30, 50, 70, 90 mg/kg	70 days	Enhanced growth performance, immunity status and intestinal health, maintained homeostasis intestinal microbiota	[3]
Rainbow trout (*Oncorhynchus mykiss*)	Microbial lysozyme	0, 500, 1000, 1500 mg/kg	60 days	Positive effects on humoral and mucosal immune defenses a	[62]
Rainbow trout (*Oncorhynchus mykiss*)	Egg white lysozyme	0, 150, 300, 450, 600 mg/kg	70 days	Enhanced growth and non-specific immune system	[61]
Nile tilapia (*Oreochromis niloticus*)	Chicken egg lysozyme	0,500, 1000,1500,3000 mg/kg	60 days	Improved growth performance, feed utilization, immunity and resistance against *Aeromonas hydrophila*	[63]
Hybrid red tilapia (*Oreochromis niloticus x O. mossambicus*)	Chicken egg white lysozyme	0, 1000, 2000, 3000 mg/kg	60 days	Improved growth performance, intestinal health, antioxidant capacity, and resistance against *Streptococcus agalactiae*	[64]
Gibel carp (*Carassius auratus gibelio*)	Chicken egg white lysozyme	0, 100, 500 and 1000 mg/kg	75 days	Modulation of intestine microbiota, intestine morphology, improved protection from pathogen	[60]
Pacific white shrimp (*Litopenaeus vannamei*)	Microbial lysozyme	0, 500, 1000, 2000 g/kg	129 days	Recommended for immunity and digestive enzymes activity modulation	[58]
Pacific white shrimp (*Litopenaeus vannamei*)	Hen egg white lysozyme	0, 5, 25, 125 625 mg/kg	84 days	Inhibition of *Vibrio*, stimulation of immune- and antioxidant-related gene expression indicating resistance against White feces syndrome	[59]

## Potential Applications in Aquaculture

The aquaculture industry is experiencing significant growth to meet the increasing global demand for high-quality protein sources. However, limitations like disease outbreaks or environmental stressors remain major challenges that compromise production efficiency and sustainability [65]. In this context, feed additives with immunomodulatory and antimicrobial potential are being explored as strategies to improve health status and enhance resilience to pathogens in farmed aquatic species.

Lysozyme, as a naturally occurring antimicrobial enzyme, has demonstrated promising results in poultry and swine production by improving growth performance, enhancing intestinal health, and reducing pathogen load. These findings have motivated interest in evaluating whether similar benefits may be achieved in fish and shrimp, particularly given the need for alternatives to antibiotics and the growing emphasis on sustainable health management in aquaculture. Dietary fungal polypeptides with lysozyme-like activity have already been shown to protect fish against pathogens, suggesting that lysozyme itself could likely play a key role in protecting fish against disease [31, 66]. Exogenous enzymes have also shown promising effects on improving digestibility; however, the precise mechanisms by which they act in aquatic animals require further research [67]. The unclear mechanisms behind the action of exogenous enzymes in aquatic animals leave room for further exploration of lysozyme’s potential contribution to nutrient digestibility and animal growth.

In fish, feed additives could improve the digestion and absorption of nutrients in the intestine [3]. Similarly, to endogenous enzymes, exogenous ones, can facilitate the breakdown of complex macromolecules into simpler compounds that can be absorbed by the microbiota [68]. Previous research in monogastric livestock indicates that lysozyme supplementation can positively influence gut microbiota, contributing to improved intestinal health. Across the available aquaculture studies, several cross-species patterns begin to emerge. Immune enhancement is the most consistently reported outcome, with improvements in humoral immunity, phagocytic activity, immune-related gene expression, and pathogen resistance observed in shrimp, trout, carp, and tilapia. Responses related to growth tend to occur at moderate inclusion levels, particularly in carp, trout, and tilapia. Evidence from shrimp, trout, and tilapia also indicates digestive benefits too, including increased digestive enzyme activity, improved intestinal morphology, and more stable microbial communities. This suggests potential roles in nutrient absorption and gut homeostasis. While the evidence base remains limited and heterogeneous, these recurring improvements in immunity, disease resistance, and indicators of gut health collectively highlight lysozyme as a promising functional additive for aquaculture. The following section outlines the existing, albeit limited, research regarding the application and effects of exogenous dietary lysozyme in aquaculture species.

## Challenges and Considerations

Although lysozyme presents a promising functional additive for aquaculture, its use raises several important challenges and considerations that require careful evaluation. It is also important to recognize that extrapolating findings from terrestrial monogastrics to aquatic species has inherent limitations, as differences in digestive physiology, enzyme stability, mucosal immunity, and exposure to waterborne pathogens may influence lysozyme’s functional performance in fish and shrimp. Currently, hen egg-white lysozyme (HEWL) is the most commercially available form of lysozyme, the main concerns of which are the high recovery cost and low activity [69]. When it comes to costs, studies reported that the production cost is around $2, with human lysozyme (HL) from transgenic rice cost $2.19/g, while the manufacturing cost for HEWL was reported as $2.05/g [70, 71]. Lysozyme can also be produced through microbial fermentation. Microorganisms such as *Aspergillus sp*., *Bacillus sp*., *Kluyveromyces sp*., and various yeasts have been utilized in the food industry for lysozyme production [72]. However, limitations related to the cost of in-vitro lysozyme production need to be addressed in this case as well [73].

Another critical aspect is the form of lysozyme used, as its source, purity, and structural integrity can significantly influence its stability, bioavailability, and biological activity. Various methods, such as direct crystallisation, ion exchange, ultrafiltration, two-phase system separation, reverse micelle extraction, and affinity membrane chromatography, have been developed for lysozyme extraction [74]. Moreover, technological and environmental factors, such as pH, temperature, salt concentration, and osmotic strength, can affect the stability and antimicrobial activity of the enzyme. This also, varies according to the lysozyme types [75, 76]. For instance, HEWL is believed to have lower stability and activity under highly acidic (pH < 3.8) and alkaline conditions [20].

Enzymatic activity is strongly affected by how long the enzyme remains active in the digestive tract, since most exogenous enzymes function optimally only within neutral to slightly acidic pH ranges. Thus, extended exposure of an exogenous enzyme to an unfavourable environment can lower its catalytic activity, due to acid denaturation and proteolytic digestion [77]. As such, the successful application of exogenous lysozyme in aquafeeds depends heavily on protecting the enzyme from early inactivation within the digestive tract of aquatic animals.

Regulatory aspects and safety evaluation are key steps in the commercialisation of lysozyme-based feed additives, too. Feed enzymes must be produced in accordance with Current Good Manufacturing Practice, e.g., contained, pure-culture fermentation of microbial strains that have been documented to be safe, and enzyme preparations must be manufactured to comply with the chemical and microbiological purity standards established by FAO/WHO and the Food Chemicals Codex [78]. Enzymes as a class are rarely toxic, while lysozyme’s acute, subacute, and chronic toxicity in animals [20, 78]. However, egg lysozyme is a known allergen that can cause adverse allergic reactions to susceptible individuals [79]. This highlights the need to implement occupational safety protocols during feed production to protect workers from potential allergen exposure.

## Conclusion

The increasing demand for aquaculture products, along with the rapid deterioration of the aquaculture ecological environment, leading to a high incidence of disease, highlights the need for functional additives that can meet the animals’ nutritional requirements while maintaining their health and welfare [6, 25].

Lysozyme is a glycosidic enzyme and bioactive protein with antimicrobial properties [71], making it a promising candidate for use as a functional additive in aquaculture. Insights from terrestrial livestock suggest that administration of exogenous lysozyme could have beneficial effects on the growth performance, the immunity, and the intestinal health and microbiota. The positive results coming from monogastric livestock research provide a strong rationale for exploring its potential application in aquaculture systems. Despite the fact that its use in aquaculture has received considerably less attention, the limited number of available studies suggests that lysozyme supplementation may result in both zootechnical advantages and immunological benefits in aquatic animals. Thus, there is still space to investigate the potential benefits of exogenous lysozyme, for example, in Mediterranean aquaculture species for which experimental data are currently lacking. However, when translating findings from monogastric livestock to aquatic species, it is crucial to consider fundamental physiological and environmental differences, which may influence lysozyme’s stability, mode of action, and overall efficacy in fish and shrimp. This highlights the importance of species-specific validation before broad implementation. Furthermore, careful consideration of the limitations is essential when evaluating novel feed additives. Although, lysozyme is rarely toxic [20, 78] appropriate controls are necessary to ensure consistent enzyme characteristics, such as stability, and to implement safety measures for user protection.

## Data Availability

No datasets were generated or analysed during the current study.
